# Disruption of the rice nitrate transporter OsNPF2.2 hinders root-to-shoot nitrate transport and vascular development

**DOI:** 10.1038/srep09635

**Published:** 2015-04-29

**Authors:** Yuge Li, Jie Ouyang, Ya-Yun Wang, Rui Hu, Kuaifei Xia, Jun Duan, Yaqin Wang, Yi-Fang Tsay, Mingyong Zhang

**Affiliations:** 1 Key Laboratory of South China Agricultural Plant Molecular Analysis and Genetic Improvement & Guangdong Provincial Key Laboratory of Applied Botany, South China Botanical Garden, Chinese Academy of Sciences, Guangzhou 510650, China; 2 Rice Institute, Chongqing Academy of Agricultural Sciences, Chongqing 401329, China; 3 Department of Life Science, National Taiwan University, Taipei, Taiwan; 4 University of Chinese Academy of Sciences, Beijing 100049, China; 5 Guangdong Key Lab of Biotechnology for Plant Development, College of Life Science, South China Normal University, Guangzhou 510631, China; 6 Institute of Molecular Biology, Academia Sinica, Taipei, Taiwan

## Abstract

Plants have evolved to express some members of the nitrate transporter 1/peptide transporter family (*NPF*) to uptake and transport nitrate. However, little is known of the physiological and functional roles of this family in rice (*Oryza sativa* L.). Here, we characterized the vascular specific transporter OsNPF2.2. Functional analysis using cDNA-injected *Xenopus laevis* oocytes revealed that OsNPF2.2 is a low-affinity, pH-dependent nitrate transporter. Use of a green fluorescent protein tagged OsNPF2.2 showed that the transporter is located in the plasma membrane in the rice protoplast. Expression analysis showed that *OsNPF2.2* is nitrate inducible and is mainly expressed in parenchyma cells around the xylem. Disruption of *OsNPF2.2* increased nitrate concentration in the shoot xylem exudate when nitrate was supplied after a deprivation period; this result suggests that OsNPF2.2 may participate in unloading nitrate from the xylem. Under steady-state nitrate supply, the *osnpf2.2* mutants maintained high levels of nitrate in the roots and low shoot:root nitrate ratios; this observation suggests that OsNPF2.2 is involved in root-to-shoot nitrate transport. Mutation of *OsNPF2.2* also caused abnormal vasculature and retarded plant growth and development. Our findings demonstrate that OsNPF2.2 can unload nitrate from the xylem to affect the root-to-shoot nitrate transport and plant development.

Nitrogen (N) is a primary nutritional element for plant growth and development. N is also a major limiting factor for crop yield^1^,^2^. However, if N fertilizers added to soils are not absorbed efficiently by plants, the fertilizers can cause serious environmental pollution. Nitrate (NO_3_^−^) and ammonium (NH_4_^+^) are two major N sources for higher plants. Like most upland plants, lowland rice uses nitrate and ammonium as its major N sources^3^. In rice, nitrate acquisition is not only of high capacity and efficiency but is also considered superior to ammonium acquisition^4^. Furthermore, the growth and yield of rice is superior on mixtures of nitrate and ammonium than with provision of either N source alone^3^. Under cultivation, the proportion of available nitrate in soils increases, and NO_3_^−^-N becomes the dominant form instead of NH_4_^+^-N, which is traditionally assumed to be the preferred N source for paddy rice^5^. Nitrate and ammonium concentrations range from less than 100 μM to more than 10 mM in soil solutions, so plant roots have uptake systems for both NO_3_^−^ and NH_4_^+^, with different affinities^6^,^7^.

Nitrate uptake and allocation are key factors in efficient N utilization for higher plants^2^,^6^. Membrane-bound transporters are required for nitrate uptake from the soil and for inter- and intracellular movement of nitrate inside the plants^6^. In *Arabidopsis*, at least five gene families are involved in the uptake, allocation, and sensing of nitrate^1^,^6^,^8^,^9^. These include the *NPF* (previously *NRT1/PTR*) family^10^ for low-affinity transport of nitrate or di/tripeptides, the *NRT2* family for high-affinity nitrate transport, the *CLC* family for chloride channels, the *SLAC1*/*SLAH* family which contains the gene encoding slow anion channel-associated 1 homolog 3, and the ALMT family for aluminum-activated malate transport.

Only a small proportion of the 53 *NPF* genes in *Arabidopsis* have been functionally characterized for nitrate transport activity^1^,^10^. Some *Arabidopsis* NPFs are also able to transport other substrates, such as auxin, abscisic acid, and glucosinolates^11^,^12^,^13^. Most of the *NPF* nitrate transporters are low-affinity nitrate transporters^1^; only AtNPF6.3 (AtNRT1.1, CHL1)^14^,^15^ and MtNPF6.8 (MtNRT1.3)^16^ are dual-affinity nitrate transporters.

Nitrate is taken up by roots from soil and transported to shoots and seeds for storage and/or further assimilation^1^. AtNPF6.3 is involved in the uptake of nitrate from the soil^14^,^17^ and as a bidirectional transporter in the translocation of nitrate from the roots to the shoots^18^. AtNPF7.3 (AtNRT1.5) is responsible for loading nitrate into the xylem for root-to-shoot nitrate transport^19^. AtNPF7.2 (AtNRT1.8) and AtNPF2.9 (AtNRT1.9) are involved in regulating root-to-shoot nitrate translocation of xylem and phloem respectively^20^,^21^. AtNPF6.2 (AtNRT1.4) regulates leaf nitrate homeostasis and leaf development, and AtNPF2.13 (AtNRT1.7) mediates phloem loading to allocate nitrate from older to younger leaves^22^,^23^. AtNPF2.12 (AtNRT1.6) is involved in delivering nitrate to developing seeds^24^.

AtNPF6.3 also functions as a nitrate sensor that regulates transcription^25^ and participates in promoting lateral root elongation in NO_3_^−^ rich medium^26^. In the absence of nitrate, AtNPF6.3 facilitates the uptake of the phytohormone auxin^13^. These studies showed that nitrate-regulated AtNPF6.3-dependent auxin transport is responsible for nitrate-promoted lateral root elongation. The high-affinity uptake complex NRT2.1-NAR2.1 also participates in regulating lateral root development^27^; the regulation of lateral root growth by NRT2.1 and NAR2.1 is independent of their uptake function^28^.

Rice is a main cereal crop that provides the staple food for more than half of the world's population. However, the efficiency of nitrogen utilization by rice is lower than that for other crops^29^, and rice varieties show genetic variation with respect to the level of nitrogen fertilization required^30^. Most of our knowledge about nitrate uptake and translocation is from the study of *Arabidopsis*, and little is known about these processes in rice^1^,^2^. Therefore, an understanding of the molecular mechanisms of nitrate uptake and translocation in rice is very important for improving the efficiency of nitrogen use in rice. In the rice genome, there are more than 93 *NPF* genes^10^,^31^,^32^, and 5 *NRT2* genes^33^,^34^ have been predicted. However, to date, only six members of the rice *NPF* gene family have been studied, and only OsNPF8.9 (OsNRT1)^35^ and OsNPF2.4^36^ have been functionally demonstrated to transport nitrate. Here, we characterized the rice *NPF* family member OsNPF2.2 and show that it is a low-affinity nitrate transporter that participates in unloading nitrate from the xylem and influences root-to-shoot nitrate transport and plant development.

## Results

### OsNPF2.2 is a low-affinity nitrate transporter

Rice *OsNPF2.2* (LOC_Os12g44100) is classified into the *NPF2* subfamily of the *NPF* family^10^. It's mRNA (accession no. AK068351) is predicted to encode a 589 amino-acid protein that has a high degree of homology with low-affinity nitrate transporters. The OsNPF2.2 protein is 58%, 45%, 41%, and 47% homologous to OsNPF2.4, AtNPF2.13, AtNPF2.12, and AtNPF2.9, respectively, all of which are involved in nitrate transport, and 48% and 50% homologous to AtNPF2.10 (AtGTR1) and AtNPF2.11 (AtGTR2), respectively, both of which are involved in glucosinolate transport. It contains 12 putative transmembrane domains (TMs) with a long hydrophilic loop between TM6 and TM7 (Supplementary Fig. S1); this structure is similar to the typical structure of NPF (NRT1/PTR) transporters^37^,^38^.

**Figure 1:**
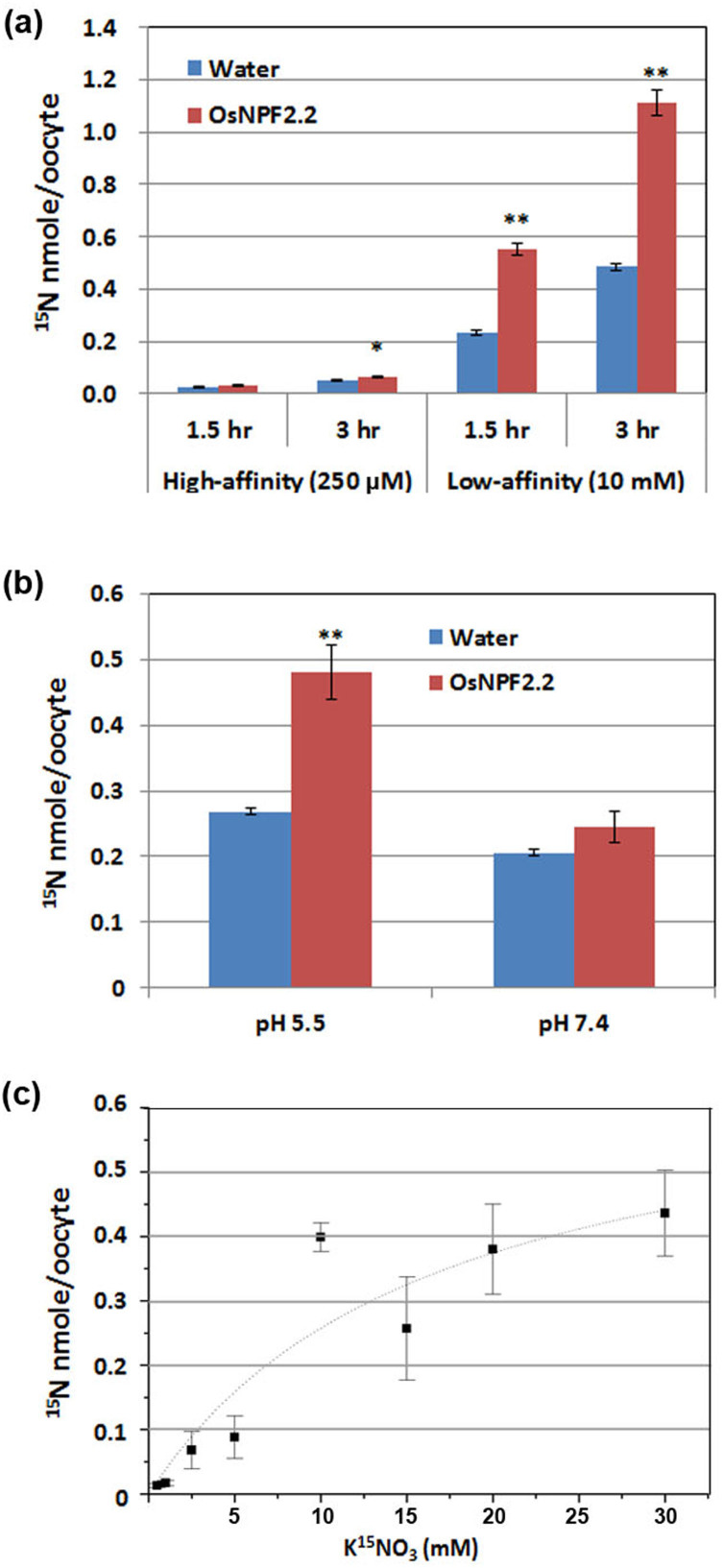
OsNPF2.2 is a low-affinity nitrate transporter. (a) Nitrate uptake activity of *OsNPF2.2*-injected oocytes at pH 5.5. High-affinity or low-affinity nitrate uptake activity was examined by incubating oocytes with 0.25 or 10 mM K ^15^NO_3_ for 1.5 h and 3 h and then measuring the levels of ^15^N in the oocytes (n = 8–13 oocytes for the water- and *OsNPF2.2*-injected oocytes, respectively). (b) The pH dependence of the nitrate uptake activity of OsNPF2.2. The *OsNPF2.2*-injected oocytes were incubated with 10 mM K ^15^NO_3_ buffered at pH 5.5 or 7.4 for 2 h, and then the levels of ^15^N in the oocytes were measured (n = 10–13 oocytes for the water- and *OsNPF2.2*-injected oocytes, respectively). (c) Uptake kinetics of OsNPF2.2. *OsNPF2.2*-injected oocytes were incubated with different concentrations of K ^15^NO_3_ at pH 5.5 for 1.5 h, and then their ^15^N contents were determined. The *K*_m_, calculated from one experiment by fitting the data to the Michaelis–Menten equation using a nonlinear least squares methods in the ORIGIN 5.0 program (Microcal Software; GE Healthcare), was ~16.6 ± 12.9 mM. The values are mean ± SE (n = 4–9 oocytes for each concentration). *P < 0.05 when compared to the water-injected control; **P < 0.01 when compared to the water-injected control.

To determine whether OsNPF2.2 is a nitrate transporter, its cDNA was heterologously expressed in *Xenopus laevis* oocytes. We assayed high- and low- affinity nitrate transport activity with 250 μM and 10 mM ^15^NO_3_^−^, respectively. As shown in Fig. 1a, as compared with water-injected oocytes, *OsNPF2.2*-injected oocytes at pH 5.5 showed significantly enhanced ^15^NO_3_^−^ uptake activity with 10 mM NO_3_^−^ after 1.5 h and 3 h of incubation. To confirm that OsNPF2.2 functioned as expected for a proton-coupled nitrate transporter, the NO_3_^−^ transport activity of *OsNPF2.2*-injected oocytes was measured at pH 5.5 and pH 7.4, respectively (Fig. 1b). The enhanced ^15^NO_3_^−^ uptake activity of *OsNPF2.2*-injected oocytes was only detected at pH5.5. To determine the affinity of OsNPF2.2 for NO_3_^−^, the uptake activity of *OsNPF2.2*-injected oocytes at pH 5.5 was measured using different concentrations, ranging from 0.5 to 30 mM, of ^15^N-labeled NO_3_^−^, and the K_m_ for NO_3_^−^, calculated by fitting the data to the Michaelis–Menten equation, was estimated as ~16.6 ± 12.9 mM (Fig. 1c). The results from the functional studies in *Xenopus* oocytes indicate that OsNPF2.2 is a pH-dependent, low-affinity nitrate transporter.

### OsNPF2.2 is localized in the plasma membrane

**Figure 2:**
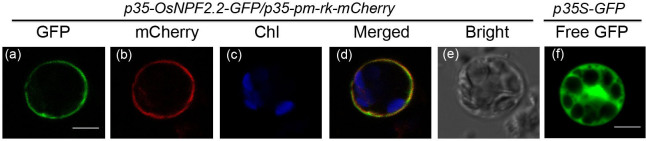
OsNPF2.2 is located in the rice plasma membrane. (a–e) Fluorescence of *OsNPF2.2-GFP* coexpressed with the plasma membrane marker pm-rk-mCherry in transiently transformed rice protoplasts (bar = 5 μm). Transformed rice protoplasts were first identified by their green fluorescent protein (GFP) fluorescence from OsNPF2.2-GFP (a); then, these cells were checked for mCherry fluorescence (b) and finally for chlorophyll autofluorescence (c). The corresponding bright-field image is shown in (e). (d) Merged images from (a), (b), and (c). (f) GFP fluorescence from free GFP expressed under the *35S* promoter as a control (bar = 5 μm).

To determine the subcellular location of OsNPF2.2, an OsNPF2.2-GFP fusion protein under the control of the CMV *35S* promoter^39^ was transiently coexpressed with the plasma membrane marker pm-rk-mcherry^40^ in rice protoplasts. Unlike the signal for free GFP (Fig. 2f), which was found in the whole cell, the signal for the OsNPF2.2-GFP fusion protein (Fig. 2a) was localized in the plasma membrane and overlapped (Fig. 2d) with the signal for the marker pm-rk-cherry (Fig. 2b). Taken together with the observation that OsNPF2.2 has 12 TMs (Supplementary Fig. S1), these data indicate that OsNPF2.2 is a plasma membrane nitrate transporter.

### *OsNPF2.2* expression is induced by exogenous nitrate

**Figure 3:**
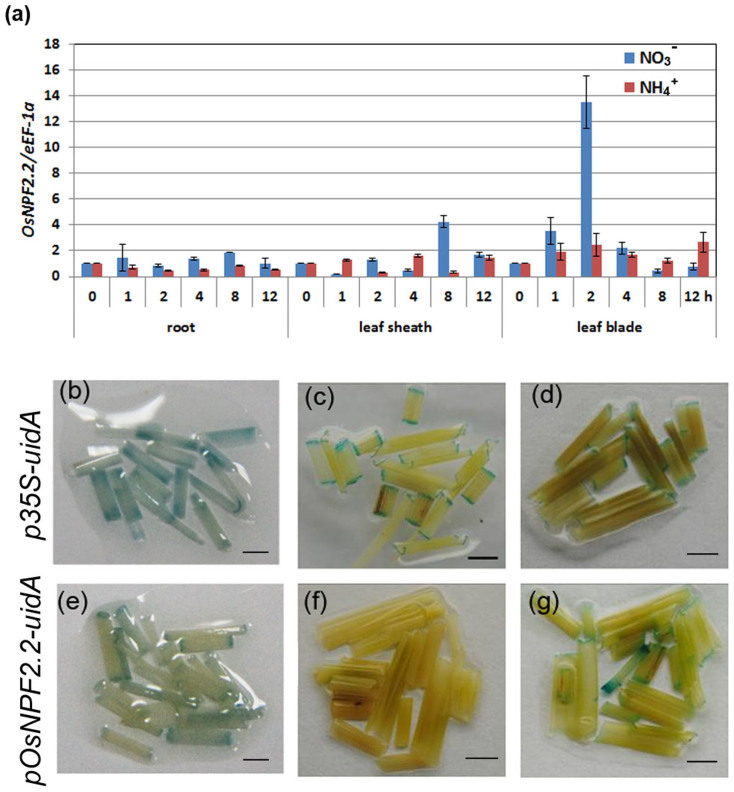
*OsNPF2.2* expression in leaf blades is nitrate inducible. (a) Time-course analysis of *OsNPF2.2* expression following NO_3_^−^ and NH_4_^+^ induction by quantitative real-time–PCR. Rice seedlings were grown on 1/2 Murashige and Skoog solid medium for 10 d; next, seedlings were washed and deprived of NO_3_^−^ for 1 d and then subcultured in International Rice Research Institute (IRRI) solution with 10 mM KNO_3_ or 10 mM NH_4_Cl as the N source, respectively, or mock-treated with KCl. The plants were collected for RNA extraction at the indicated times. Relative expression was normalized to the expression level of *eEF-1a*. The values are mean ± SE for triplicate samples, with each replicate containing seven seedlings. (b–g) β-Glucuronidase activity analysis in leaf blades of the transgenic rice plants harboring the *uidA* gene driven by the cauliflower mosaic virus *35S* promoter (b–d) or the 2.5 kb *OsNPF2.2* promoter (e–g) after nitrate induction. The transgenic plants were first grown in 1.4 mM NH_4_NO_3_ solution (b, e) and then deprived of NO_3_^−^ for 1 d (c, f); finally, the plants were transferred back into IRRI solution with 10 mM KNO_3_ as the N source (d, g) for 2 h. Bar = 0.25 cm.

*Arabidopsis* *NPF* genes are expressed differently in response to nitrate supply and N-starvation^2^. Transporters are also involved in the sensing of nitrate^9^. We therefore performed qPCR to check the transcriptional response of *OsNPF2.2* to nitrate and ammonium treatment (Fig. 3a). *OsNPF2.2* expression was upregulated in leaf blades in response to 1–2 h of 10 mM nitrate resupply after 1 d nitrate deprivation (Fig. 3a). Moreover, in the roots and the leaf sheaths, *OsNPF2.2* expression was slightly induced by 10 mM nitrate resupply. In contrast, in all the tissues tested, *OsNPF2.2* expression was not significantly affected by NH_4_^+^ treatment (Fig. 3a). To further confirm that nitrate upregulated *OsNPF2.2* expression in leaf blades, we examined GUS staining in *pOsNPF2.2*-*uidA* transgenic rice after NO_3_^−^ induction (Fig. 3e–g). Before NO_3_^−^ deprivation, GUS staining was present in the leaves of both *pOsNPF2.2*-*uidA* and *p35S-uidA* transgenic rice plants (Fig. 3b, e). However, when the plants were transferred into an NO_3_^−^-deprived solution for 1 d, GUS staining disappeared from the leaves of the *pOsNPF2.2*-*uidA* plants (Fig. 3f) but remained in the leaves of the *p35S-uidA* plants (Fig. 3c). Then, when the plants were transferred back the NO_3_^−^ solution, the GUS staining appeared in the leaves of the *pOsNPF2.2*-*uidA* plants (Fig. 3g). These results show that *OsNPF2.2* expression is induced by exogenous nitrate, or repressed by nitrate starvation, different from *OsNPF2.4*^36^ which was induced by N starvation in the older leaf blades.

### *OsNPF2.2* is mainly expressed in parenchyma cells of the vasculature

**Figure 4:**
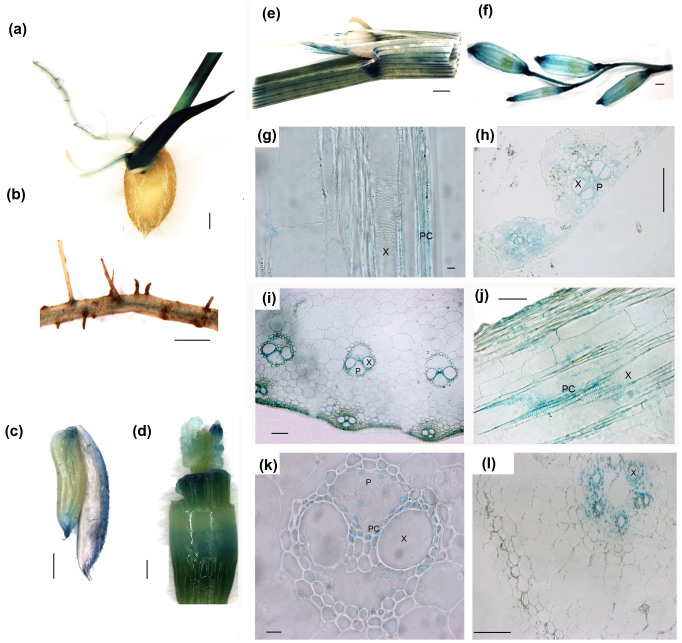
*OsNPF2.2* is strongly expressed in parenchyma cells around xylem vessels. (a–f) *OsNPF2.2* expression patterns in various organs by β-glucuronidase staining analysis, bar = 1000 μm. The transgenic plants harboring the *uidA* gene driven by the 2.5 kb *OsNPF2.2* promoter were grown in a paddy with normal nitrogen fertilizer. GUS activity was detectable in the roots and leaves of germinating seeds (a), in old roots (b), in filling seeds (c), in young inflorescences and stems (d), in flag leaves (e), and in panicles (f). (g–l) Longitudinal (g, j) and transverse (h, i, k, and l) section analysis of GUS-stained plants transgenic for *pOsNPF2.2-uidA* and grown under normal nitrate conditions. GUS staining revealed that *OsNPF2.2* is strongly expressed in vascular parenchyma cells in leaves (g, h), stems (i, j, k), and roots (l). X, xylem; P, phloem; PC, parenchyma cell. Bar = 5 µm in (g), 100 µm in (h), 50 µm in (i, j and l), and 10 µm in (k).

Three approaches were used to examine the expression pattern of *OsNPF2.2*. First, the microarray data from RiceXPro (http://ricexpro.dna.affrc.go.jp/) showed that *OsNPF2.2* is mainly expressed in leaves, roots, stems, inflorescences, anthers, pistils, lemmas, paleas and ovaries (Supplementary Fig. S2). Second, whole-mount GUS staining showed that although *OsNPF2.2* is expressed in all the organs examined (Fig. 4a–f), it is preferentially expressed in the central stele in roots (Fig. 4a, b) and in leaf veins (Fig. 4e). Third, to determine the tissue-specific expression pattern of *OsNPF2.2*, cross- or longitudinal sectioning of the GUS-stained organs was performed (Fig. 4g–l). Sectioning confirmed that *OsNPF2.2* is mainly expressed in leaf veins (Fig. 4g, h), stem vasculature (Fig. 4i, j, and k), and in the stele in roots (Fig. 4l). However, strong GUS staining was observed in vascular parenchyma cells (Fig. 4g–i, 4l). Transverse and longitudinal sectioning of the stems and the leaves revealed that *OsNPF2.2* is expressed in the parenchyma cells abutting xylem vessels (Fig. 4g, j, and k). Taken together with the previous results, these observations show that *OsNPF2.2* is strongly expressed in the parenchyma cells of vasculature in response to nitrate application.

### Disruption of *OsNPF2.2* hinders plant growth and seed filling

**Figure 5:**
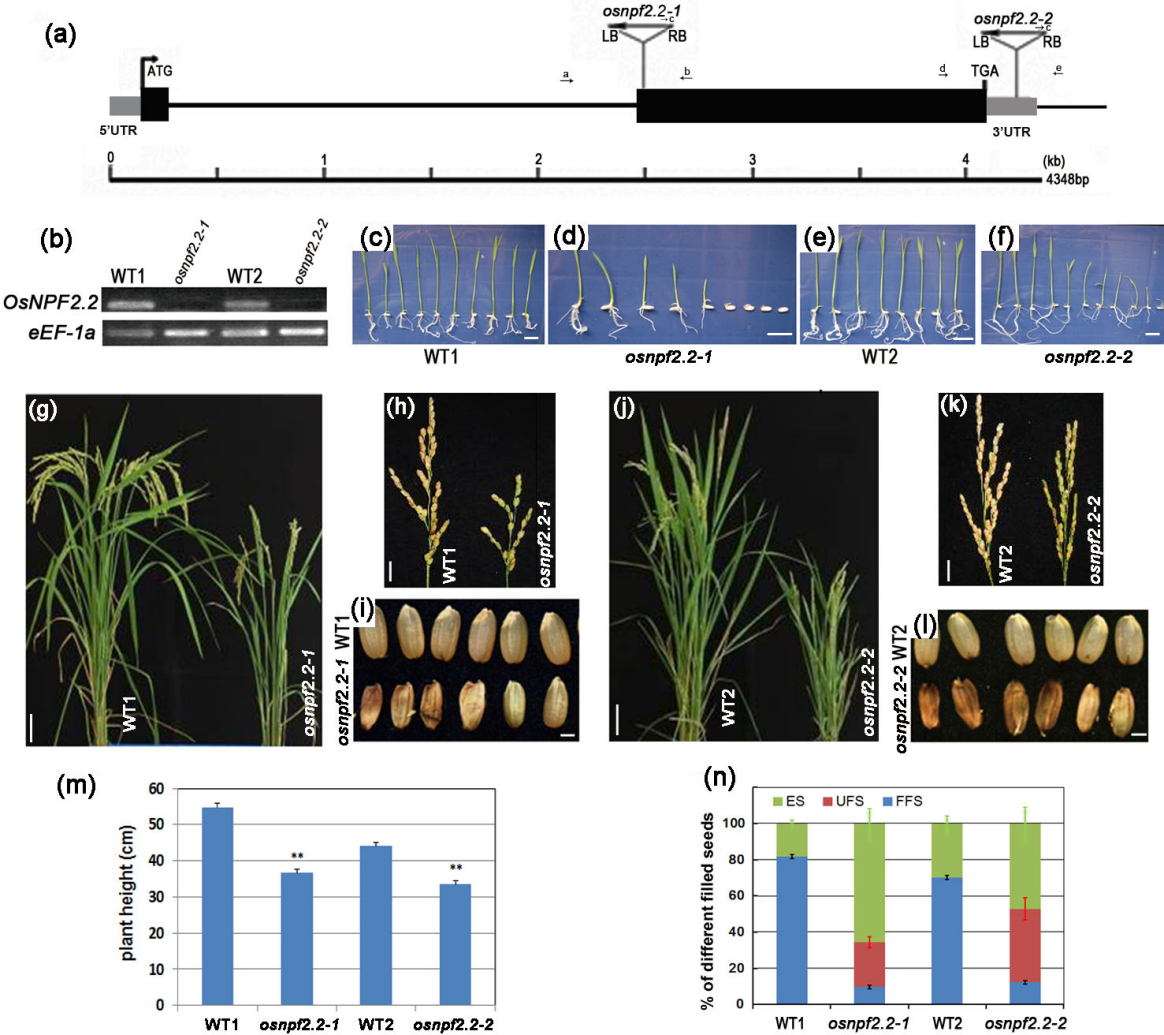
Disruption of *OsNPF2.2* hinders plant growth and seed filling. (a) Schematic diagram of *OsNPF2.2* and insertion positions of transfer DNA (T-DNA) in the *osnpf2.2-1* and *osnpf2.2-2* mutants. The boxes indicate two exons, and the connecting black line represents the intron. Black boxes indicate the open reading frame, and the gray boxes indicate the 5′- and 3′-untranslated regions. The T-DNA is indicated by the triangle. The abbreviations LB and RB indicate the left and right borders, respectively, of the T-DNA. Arrows with letters show the locations of the primers used to amplify the flanking sequence tags and screen the homozygous mutants. (b) Semi-quantitative–PCR analyses of *OsNPF2.2* expression in *osnpf2.2* mutants. Total RNA was extracted from the leaves of homozygous *osnpf2.2-1* and *osnpf2.2-2* plants and their parental (WT) varieties. The gene *eEF-1a* was amplified as an internal control. (c–f) Seed germinating comparison between the *osnpf2.2* mutants and WT plants. The surface-sterilized seeds were germinated on 1/2 Murashige and Skoog solid medium; bar = 2 cm. (g–n) Main phenotypes of the *osnpf2.2* mutants grown in a paddy with normal nitrogen fertilizer. (g, j) Dwarf plants; bar = 5 cm; (h, k) Short panicles; bar = 1.5 cm; (i, l) Reduction in filling for seeds from the *osnpf2.2* mutants as compared to WT seeds; bar = 2 mm. (m) Plant height statistics of the *osnpf2.2* mutants and WT plants at the mature stage; **P < 0.01 when compared to WT plants. (n) Comparison analysis of the fully filled seeds (FFS), the unfilled seeds with expanded ovaries (UFS), and the empty spikelets (ES) per plant between the *osnpf2.2* mutants and WT plants. The values are mean ± SE from 60 plants at two seasons.

To investigate the function of *OsNPF2.2*, we identified two *japonica* rice (*Oryza sativa* L.) mutants, *osnpf2.2-1* (3D-02162) and *osnpf2.2-2* (3A-07557), which were T-DNA knockouts developed from the Hwayoung and Dongjin varieties, respectively^41^,^42^. Sequencing of the genomic fragment flanking the T-DNA insertion sites in the two mutants confirmed that the T-DNA was inserted in the *OsNPF2.2* coding region in *osnpf2.2-1* (3D-02162), and in the *OsNPF2.2* 3′-untranslated region in *osnpf2.2-2* (3A-07557; Fig. 5a). T-DNA copy number analysis showed that there was a single T-DNA locus in *osnpf2.2-1*, and two in *osnpf2.2-2* (data not shown). *OsNPF2.2* transcripts were not detected in either mutant (Fig. 5b). Since most of the seeds from the two *osnpf2.2* mutants were abnormal, we failed to obtain complemented lines from the mutants. However, a few homozygous mutants were identified by PCR screening and used for analysis. Homozygous *osnpf2.2-1* and *osnpf2.2-2* mutants had similar phenotypes in our analysis. In addition, to validate results from the two *osnpf2.2* mutants, we also generated *OsNPF2.2* knockdown mutants by RNAi under the control of the rice *OsNPF2.2* promoter. We found that eight independent *OsNPF2.2*-RNAi transgenic lines exhibited a reduction in *OsNPF2.2* transcript level (Supplementary Fig. S3a).

The *osnpf2.2* mutants displayed several phenotypic alterations throughout their lifespans when grown under normal N condition (Fig. 5c–5n). Unlike wild-type seeds, most of the seeds from the *osnpf2.2* mutants did not germinate on wet tissues. When germinated on 1/2 MS medium, the seed germination rates for *osnpf2.2-1* and *osnpf2.2-2* were obviously lower than that of their parental varieties (Fig. 5c–f). The *osnpf2.2* mutant plants were dwarf (Fig. 5g, j, and m), and the panicles were short (Fig. 5h, k). Grain filling in the *osnpf2.2* mutants was severely blocked (Fig. 5n). The altered phenotypes of the two *osnpf2.2* mutants were similar, even though the *osnpf2.2-2* mutant had two copies of T-DNA. Growth retardation was also observed in the mutant plants when grown under low or high nitrate application (Supplementary Fig. S4). Most of the *osnpf2.2* mutant phenotype was seen in the *OsNPF2.2*-RNAi transgenic plants, including dwarfism, short panicles, and low seed setting (Supplementary Fig. S3b–f). Based on the above data, we concluded that the phenotypic alterations observed in the *osnpf2.2* mutants were caused by the mutations in *OsNPF2.2*. Therefore, we used homozygous mutant, *osnpf2.2-1* and *osnpf2.2-2* plants for further analysis.

Next, we carefully checked seed development in the *osnpf2.2* mutants (Fig. 5n). To determine whether the low rate of seed setting in the *osnpf2.2* mutants is caused by semisterile pollen or fertilized but aborted ovaries, we counted the number of the fully filled seeds, unfilled seeds with expanded ovaries (UFS), and empty spikelets per plant (Fig. 5i, l). The data show that, for every mutant plant, the percentage of UFS is much higher than for the wild-type plant (Fig. 5n). UFS are seeds that contain fertilized egg cells but were unfilled. In addition, even the fully filled seeds of the mutants may be abnormal, since they did not germinate normally (Fig. 5c–f). Therefore, the disruption of *OsNPF2.2* severely affects the development of rice seeds.

Although the two *osnpf2.2* mutants and the *OsNPF2.2*-RNAi rice showed similar phenotypes, some differences were also observed (Fig. 5, Supplementary Fig. S3). These differences might be caused by differences in the genetic background of the two varieties and/or the different placement of the T-DNA insertion sites (Fig. 5a).

### Effect of *OsNPF2.2* on root-to-shoot nitrate transport

**Figure 6:**
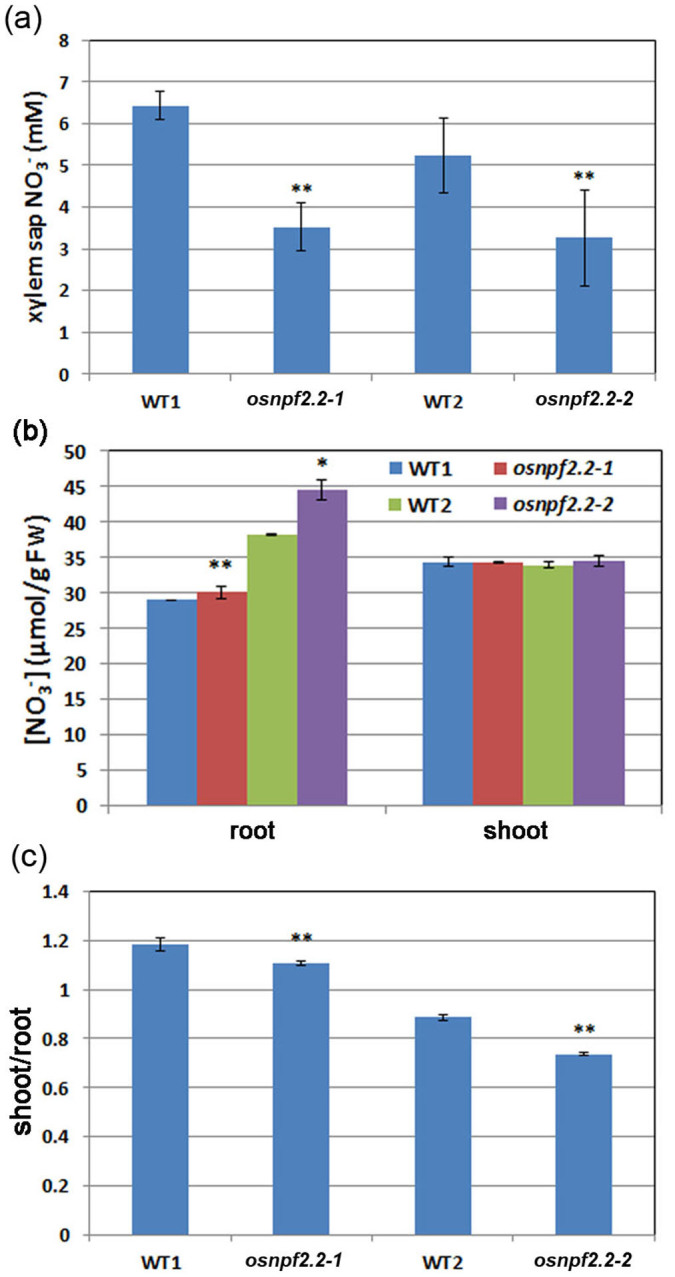
Disruption of *OsNPF2.2* affects nitrate transport from root to shoot. (a–c) Nitrate concentrations in the xylem exudate (a), total nitrate concentration in the roots and shoots (b), and the shoot:root nitrate ratio (c) of the wild-type (WT) plants and the *osnpf2.2* mutants. The plants were grown in International Rice Research Institute (IRRI) solution containing 1.4 mM NH_4_NO_3_ for 8 weeks for the steady-state nitrate treatment. To collect the xylem sap, the plants were cut 4 cm above the roots, and the roots were immediately transferred to 10 mM nitrate IRRI solution for 2 h. Xylem sap was collected over the 2 h period. To measure the total nitrate concentration in the roots and shoots, the samples were directly harvested after cultivation. The values represent mean ± SE for triplicate samples; *P < 0.05 when compared to WT plants; **P < 0.01 when compared to WT plants.

To investigate whether the *osnpf2.2* mutants are defective in long-distance transport of nitrate, the nitrate concentration in the xylem exudates, the total nitrate concentration, and the distribution of nitrate between roots and shoots were analyzed (Fig. 6). When plants were continuously grown with sufficient nitrate solution, the nitrate concentrations of the xylem sap in the *osnpf2.2* mutants were significantly lower than those in the wild-type plants (Fig. 6a). In addition, more nitrate accumulated in the roots of the mutants than in the roots of the wild-type plants (Fig. 6b), and although the shoot:root nitrate ratio was 1.18 or 0.89 in the wild-type plants, it was reduced to 1.10 or 0.73 in *osnpf2.2-1* and *osnpf2.2-2* mutant plants, respectively (Fig. 6c). These observations show that in *osnpf2.2* mutants, more nitrate is retained in the roots and less nitrate is transported into the shoots than in wild-type plants. Taken together, these results suggest that OsNPF2.2 plays a role in transporting nitrate from the roots to the shoots.

### Disruption of *OsNPF2.2* affects unloading nitrate from xylem

**Figure 7:**
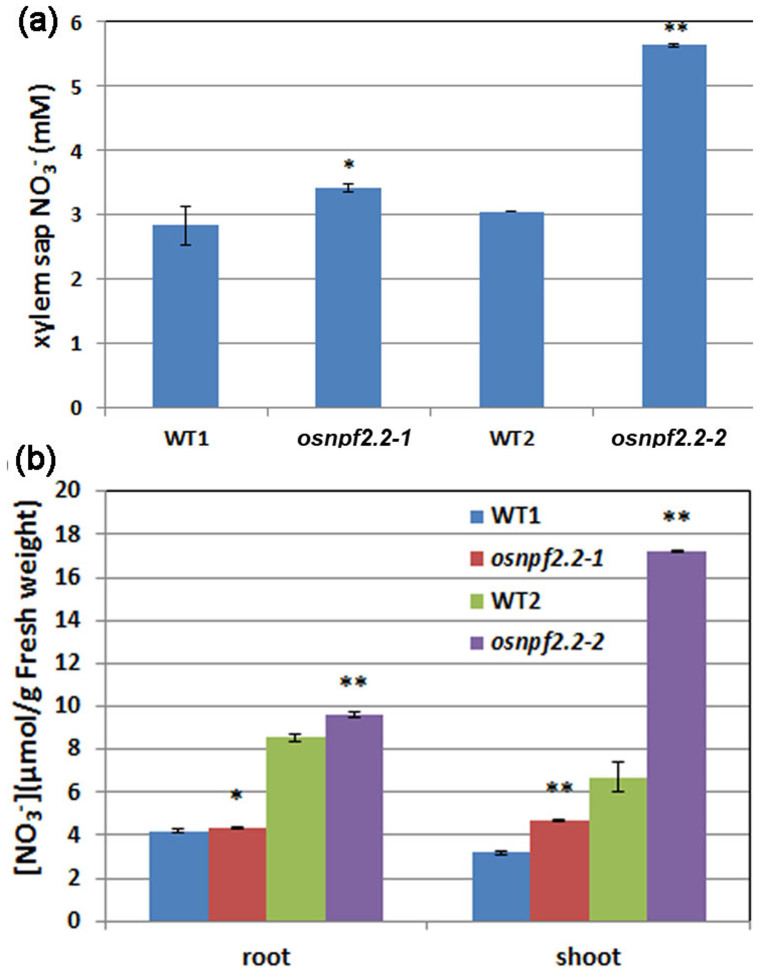
Disruption of *OsNPF2.2* affects nitrate unloading from the xylem. (a, b) Nitrate concentrations in the xylem exudates (a), and total nitrate concentration in the roots and shoots (b) of wild-type (WT) plants and the *osnpf2.2* mutants under 2 h short-term nitrate feeding. The plants were grown in International Rice Research Institute (IRRI) solution containing 1.4 mM NH_4_NO_3_ for 8 weeks; next, they were transferred to IRRI solution containing 1.4 mM NH_4_SO_4_ for NO_3_-starvation for 1 week and then transferred back to IRRI solution with 10 mM KNO_3_ for 2 h for xylem sap collection (a) or for measuring the total nitrate concentration in the roots and shoots (b). The values are mean ± SE for triplicate samples; *P < 0.05 as compared to WT plants; **P < 0.01 as compared to WT plants.

Since *OsNPF2.2* is expressed in the parenchyma cells abutting the xylem, it may be involved in nitrate loading into the xylem, like AtNPF7.3^19^, or nitrate unloading from the xylem, like AtNPF7.2^20^; therefore, disruption of *OsNPF2.2* should affect the accumulation of nitrate in the xylem sap. To test this hypothesis, the xylem exudates of plants were collected and analyzed (Fig. 7a) after a 2 h nitrate feeding following NO_3_^−^-deprivation, as follows. Plants grown in rich nitrate solution were transferred to NO_3_^−^-depleted solution (with NH_4_SO_4_ as the N source) for 1 week to remove internal nitrate. Then, the NO_3_^−^-starved plants were transferred into 10 mM nitrate IRRI solution for 2 h, after which the xylem exudates from shoots with roots were collected. The nitrate concentration in xylem sap was greater in the *osnpf2.2* mutants than in the wild-type plants (Fig. 7a). These data indicate that under short-term nitrate resupply after NO_3_^−^-starvation, more nitrates is accumulated in the xylem vessels of the *osnpf2.2* mutant than in those of the wild-type plant, suggesting that OsNPF2.2 may also play a role in unloading nitrate from the xylem. Next, the root and shoot nitrate contents of plants that had been fed with nitrate for 2 h were measured. The nitrate concentrations in the roots and the shoots of the *osnpf2.2* mutants were greater than those in the wild-type plant (Fig. 7b); this observation indicated that more nitrate was retained in roots and shoots in the *osnpf2.2* mutants than in the wild-type plant. The reason for this result may be that less nitrate is unloaded from the xylem for assimilation in the roots and the shoots under short-term nitrate feeding after NO_3_^−^-starvation in the mutant plants than in the wild-type plant. Taken together with the observation of *pOsNPF2.2*-GUS staining in the parenchyma cells of the vasculature (Fig. 4), these results suggest that disruption of *OsNPF2.2* may block nitrate unloading from the xylem.

### The *osnpf2.2* mutants display abnormal vasculature

**Figure 8:**
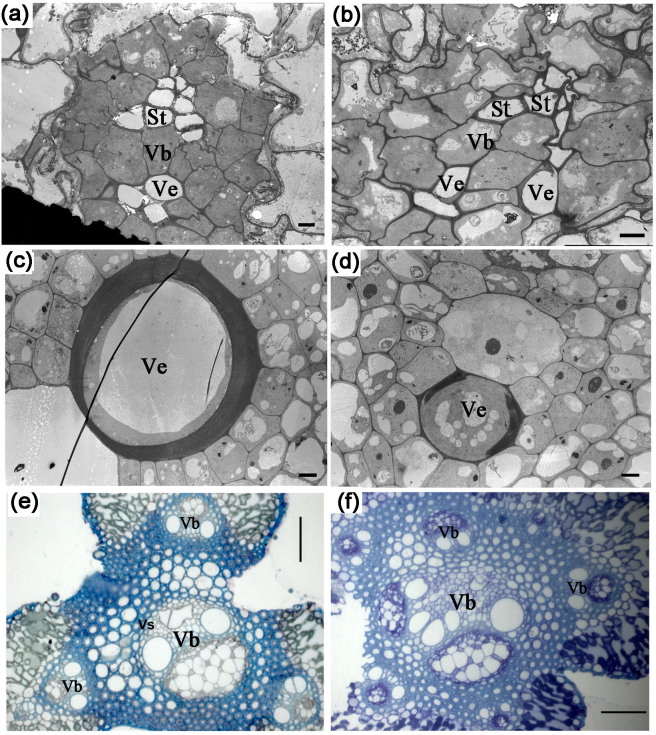
Disturbed vasculatures in the *osnpf2.2-1* mutants, as shown by transmission electron microscopy and semisection analysis. (a, b) Anther cross section from a wild-type (WT) plant (a) and an *osnpf2.2-1* mutant (b); the sieve tubes (St) and vessels (Ve) in the *osnpf2.2-1* mutant are disrupted; bar = 2 µm. (c, d) Ultrastructure of leaf blades from a WT plant (c) and an *osnpf2.2-1* mutant (d); a few vessels in the venation in the mutant plant showed delayed dying as compared with those in the WT plant; bar = 5 µm. (e, f) Semisection of primary branches from a WT plant (e) and an *osnpf2.2-1* mutant (f), bar = 50 µm. There was no obvious vascular sheath around the vascular bundle (Vb) in the *osnpf2.2-1* mutant, and its sieve tubes and vessels were irregularly distributed in the vascular bundles.

*OsNPF2.2* is highly expressed in the vasculature of all organs (Fig. 4), and the xylem and the phloem in the vasculature are important for nitrate transport^2^. To determine whether mutations in *OsNPF2.2* affect vascular development, we examined the vasculature in *osnpf2.2* mutants by sectioning. All the tested organs displayed abnormal vascular bundles (Supplementary Fig. S5). For example the external vascular bundles in the stems of the *osnpf2.2* mutants displayed abnormal structure (Supplementary Fig. S5b, d) as compared to those in wild-type plants, in which stems contain rings of regular external vasculatures (Supplementary Fig. S5a, c). The anthers and branches in *osnpf2.2-1* mutant plants also had disturbed sieve tubes and vessels (Fig. 8b, f). When examining the ultrastructure of the *osnpf2.2* mutants, we found that there was still some cytoplasm in a few vessel cells in the *osnpf2.2-1* leaves (Fig. 8d); this observation indicated that programmed cell death in some xylem vessels was blocked. From the sections, we concluded that disruption of *OsNPF2.2* disturbs vasculature development and thus affects the long-distance transport of nitrate and other metabolites.

## Discussion

The sustainable production of food to feed the world's population is critical for both human and environmental health, and the study of plant membrane transporters may be useful for enhancing the yields of staple crops^43^. In the large NPF transporter family, a few members have been functionally characterized as transporters for nitrate, peptides, glucosinolates, indole-3-acetic acid, and abscisic acid^10^,^18^; however, most of the data about these transporters has been obtained from studies in *Arabidopsis*. Rice is one of the most important cereals^30^, and it expresses 93 NPF family members^10^. However, only OsNPF8.9 and OsNPF2.4 is known to transport nitrate^35^,^36^ and physiological role of OsNPF8.9 in rice is still unknown. We showed here that OsNPF2.2 is a low-affinity nitrate transporter by expressing *OsNPF2.2* in *Xenopus laevis* oocytes, and by showing that a loss-of-function mutation for *OsNPF2.2* resulted in the accumulation of nitrate in the xylem, the reduction of nitrate transport from roots to shoots, and retardation of rice growth. Taken together with the observation that *OsNPF2.2* is expressed in parenchyma cells around xylem vessels, the data indicates that OsNPF2.2 unloads nitrate from the xylem to transport nitrate from the roots to shoots.

The rice genome contains 93 NPF members, which is more than that of *Arabidopsis*^10^. In our prior study, we found that *OsNPF2.2* expression is nitrate inducible and that OsNPF2.2 could not transport peptides^44^; mutation of *OsNPF2.2* caused serious abnormal growth. These data indicated that OsNPF2.2 might be important for rice nitrate transport. Therefore, in this study, *OsNPF2.2* was analyzed by expression in *Xenopus laevis* oocytes to test its nitrate transport capacity. In *Xenopus laevis* oocytes, OsNPF2.2 showed uptake ability for nitrate at high nitrate levels, and this nitrate uptake activity was pH dependent (Fig. 1). The various *Arabidopsis* NPF nitrate transporters function as influx, efflux, or bidirectional nitrate transporters^18^. Our data show that OsNPF2.2 has nitrate influx capacity, but we do not know whether it also has nitrate efflux capacity. *OsNPF2.2* is clustered in the *NPF2* subfamily of the *NPF* family^10^. This clade also contains AtNPF2.10 and AtNPF2.11, which are glucosinolate transporters^11^. Therefore, it will be important to further analyze whether rice contains glucosinolates, and whether OsNPF2.2 can transport glucosinolates.

Transporters for nitrate loading into xylem vessels and unloading from xylem vessels were previously identified in *Arabidopsis*^2^,^19^,^20^. In the present study, we found that *OsNPF2*.2 is strongly expressed in the parenchyma cells of the vasculature (Fig. 4) and that disruption of *OsNPF2.2* led to nitrate accumulation in the xylem sap of *osnpf2.2* mutants (Fig. 7a) when nitrate is resupplied after NO_3_^−^-starvation. When nitrate was resupplied after N-starvation, the nitrate concentration in the roots and the shoots of the wild-type plants remained at a relatively low level (<10 μM; Fig. 7b) as compared with the relatively high level of (>25 μM; Fig. 6b) seen under the steady rich nitrate condition. The reason for this observation may be as follows: when nitrate was resupplied after NO_3_^−^-starvation, the relative low nitrate level inside the wild-type plant may have caused most of the nitrate to be unloaded from the xylem vessels and be assimilated in the roots, so that less nitrate was transported to the shoot xylem. Thus, the nitrate level in the shoot xylem exudate would be low (Fig. 7a). In contrast, in *osnpf2.2* mutants under the same conditions, nitrate would have been insufficiently unloaded from the xylem and thus nitrate would have accumulated in the xylem sap, roots, and shoots of the *osnpf2.2* mutants (Fig. 7). Together with the nitrate transport activity of OsNPF2.2, these observations indicate that OsNPF2.2 is responsible for unloading nitrate from the xylem to the parenchyma cells.

However, when nitrate was resupplied after NO_3_^−^-starvation or under steady rich nitrate conditions, the changes in xylem sap nitrate concentrations of the *osnpf2.2* mutants were opposite to those seen in the wild-type plants (Fig. 6a vs. 7a). Therefore, which data could be used to explain the xylem loading and unloading? When plants are grown in low external nitrate concentration, most of the nitrate assimilation occurs in the root, whereas, under high external nitrate concentrations, shoot nitrate assimilation becomes increasingly important^45^. However, to determine the effect of OsNPF2.2 on the distribution of nitrate among organs, its function should be further investigated under the steady rich nitrate condition.

Plants adjust their development in relation to the availability of nutrient sources^46^. Although *OsNPF2.2* is mainly expressed in the vasculature (Fig. 4), the disruption of *OsNPF2.2* led to high nitrate concentrations in the roots of the *osnpf2.2* mutants (Fig. 6b) under normal steady rich nitrate conditions. However, the shoot:root ratio (Fig. 6c) of nitrate decreased in the *osnpf2.2* mutants when the plants were grown continuously under normal nitrate conditions. This observation suggests that OsNPF2.2 regulates the nitrate allocation from roots to shoots. The inhibition of nitrate transport into the leaf blades and the developing seeds in the *osnpf2.2* mutants might explain their slow growth rate and the failure of seed filling (Fig. 5 and Supplementary Fig. S4). Nitrate uptake and transfer inside plants is regulated not only by external nitrate but also by intrinsic factors^2^. High nitrate concentrations inside the *osnpf2.2* mutant plants may repress the nitrate uptake pathway via negative feedback, as occurs in the regulation of proteins such as OsNPF8.9^35^, and OsNAR2.1^47^, etc. Under nutrient limitation, an immediate response is upregulating specific transporters for nutrient uptake^48^,^49^. *OsNPF2.2* expression is induced by nitrate (Fig. 3), and it is also involved in the response to nitrate in rice. The *osnpf2.2* mutants had more serious abnormal phenotypes (Fig. 5) than the *sp1* mutant, for which the main phenotype was short panicles^50^. The *OsNPF2.2* knockout mutants and the *OsNPF2.2*-RNAi transgenic plants had similar phenotypes (Fig. 5 and Supplementary Fig. S3). These results indicate that OsNPF2.2 is important for nitrate allocation within rice plants, and for rice normal growth.

OsNPF2.2 is also involved in the development of vascular bundles (Fig. 8 and Supplementary Fig. S5). The vascular system of the plant is a network of cells that interconnects all major plant organs. Vascular tissue formation in plants is a process with broad developmental and physiological consequences. *Arabidopsis* AtNPF6.3 may be a nitrate sensor that regulates root development^13^. Disruption of *OsNPF8.20* (*OsPTR9*) caused the loss of some external stem vasculatures^51^. However, the abnormal vascular bundles in *osnpf2.2* mutants are different from those in the *osnpf8.20* mutant, as the vascular bundles in the *osnpf2.2* mutants (Fig. 8 and Supplementary Fig. S5) were much more seriously disrupted than those in the *osnpf8.20* mutant^51^. In the stems of the *osnpf2.2* mutants, all the external vasculature had an abnormal structure (Supplementary Fig. S5). The abnormal vascular bundles in the *osnpf2.2* mutants appeared in all organs, including roots, sheaths, leaves, stems, panicle branches, and anthers (Supplementary Fig. S5). In particular, some xylem cells were not correctly broken up in the *osnpf2.2* mutants (Fig. 8d). Taken together, these results indicate that OsNPF2.2 also affects the normal development of the vascular bundles. The abnormal vascular bundles in *osnpf2.2* mutants might also hinder the transport of nitrate and other metabolites. Of course, the abnormal vascular bundles might themselves be caused by the high nitrate accumulation in the *osnpf2.2* mutants.

In *Arabidopsis*, several low-affinity nitrate transporters (AtNPF6.3–AtNPF2.9) have been shown to play various roles in nitrate transport in different parts of the plant^2^. Unlike *OsNPF8.9*, which is constitutively expressed in the most external layer of the roots, that is, the epidermis and root hairs^35^,^36^, *OsNPF2.2* is constitutively expressed in most organs, even in the stele of old roots (Fig. 4), and it is induced by nitrate (Fig. 3). In addition, individual mutation of *OsNPF2.4*^36^ and *OsNPF2.2* (Fig. 5) could cause obvious phenotype. These suggest that the roles of OsNPF8.9, OsNPF2.4 and OsNPF2.2 are different in rice, while the three OsNPFs could transport nitrate. *OsNPF2.2* is highly expressed in leaves and branches (Fig. 4 and Supplementary Fig. S1); this observation suggests that OsNPF2.2 regulates nitrate transport into leaf blades and the reproductive organs.

Nitrate can be accumulated in seeds and thus can affect dormancy in seeds^52^, including rice seeds^53^. In *Arabidopsis*, AtNPF2.12^24^ and NRT2.7^54^ mediate nitrate movement into seeds. *OsNPF2.2* is highly expressed in reproductive organs and the young inflorescence (Fig. 4), and *osnpf2.2* mutants have a high percentage of unfilled seeds (Fig. 5 and Supplementary Fig. S3). These results indicate that *OsNPF2.2* has an important role in controlling nitrate movement into the spikelet and affects the development of the reproductive organs. The primary and secondary branches are main routes for nitrate transport into the spikelet, and *OsNPF2.2* is highly expressed in the panicle branches (Fig. 4). In addition, the *osnpf2.2* mutants had abnormal branches with disturbed vasculatures (Fig. 8 and Supplementary Fig. S5). These results suggest that OsNPF2.2 may control nitrate transport into the spikelet.

## Methods

### Plant materials, growth conditions

The *japonica* rice (*Oryza sativa* L.) mutants, *osnpf2.2-1* (3D-02162) and *osnpf2.2-2* (3A-07557), were transfer DNA (T-DNA) knockouts developed from the Hwayoung and Dongjin varieties, respectively, by the Korean Plant Functional Genomics Laboratory^41^,^42^. The other transgenic rice cultivar used in this study is the *japonica* rice variety Zhonghua 11. For hydroponic growth, plants were grown in a 20 L hydroponic box with an International Rice Research Institute (IRRI) rice nutrient solution^55^ in a growth rooms (28 ± 2°C, 14 h light, 10 h dark). For soil-grown experiments, plants were grown in a rice paddy of the South China Botanical Garden, Chinese Academy of Sciences, Guangzhou, China (SCBG).

### Functional analysis of *OsNPF2.2* in *Xenopus laevis* oocytes

The 1.8 kb *OsNPF2.2* cDNA was cloned into the oocyte expression vector pGEMHE and linearized with *Nhe*I. Capped mRNA was transcribed in vitro using mMESSAGE mMACHINE kits (Amion). Oocytes were isolated and injected with 50–100 ng of *OsNPF2.2* cRNA in 50 nL of water; other processes were as described previously^21^.

### Construction of vectors and plant transformation

For the *OsNPF2.2* promoter-β-glucuronidase construct, the 2.5 kb promoter of the *OsNPF2.2* gene was amplified by PCR. The following primers were used for the amplification: 5′- CCCAAGCTTATGAACAGTTGAGAAGAC-3′ (forward) and 5 ′- CATGCCATGGATTTATGTAAGATTAGGC-3′ (reverse). The resulting PCR fragment was inserted in the *Hind*III/*Noc*I sites at the 5 ′ end of the *uidA* reporter gene in pCAMBIA1301 (http://www.cambia.org).

To generate the hairpin RNAi construct, a highly specific 114 bp fragment of *OsNPF2.2* was amplified by PCR using the forward primer O25F (5′-GGTACCACTAGTCTAATCTTACATAAAT-3′), and the reverse primer O25R (5′-GGATCCGAGCTCGAGATGTGCTGCTGCTTC-3′). The resulting PCR fragment was cloned in the sense orientation into the *Spe*I/*Sac*I sites of pTCK303^56^ to generate pTCK-114i and then, cloned in the antisense orientation into the *Kpn*I/*BamH*I sites of pTCK-114i to obtain the *OsNPF2.2*-RNAi construct. Finally, the *OsPTR2* promoter was inserted into the *Hind*III/*BamH*I sites of the *OsNPF2.2*-RNAi construct, replacing the *UBI-1* promoter, to obtain the final vector (pTCK-*pOsNPF2.2*-114bp-*OsNPF2.2* Sense-Intron-114bp-*OsNPF2.2* Antisense). The construct was transformed into *Agrobacterium tumefaciens* (strain EHA105) and used to transform calli developed from Zhonghua 11 seeds to obtain the transgenic rice, as previously described^51^.

To generate a green fluorescent protein (GFP)-tagged OsNPF2.2 fusion protein construct (*p35S-OsNPF2.2-GFP*) for transient expression, *OsNPF2.2* cDNA was amplified by PCR using the forward primer 5′- GGATCCGCCTAATCTTACATAAAT-3′ and the reverse primer 5′- GGATCCGACCGCCATGGCCGGCGG-3′. The amplified fragment was then subcloned in front of the *GFP* coding region in the vector pUC18-GFP.

### Semi-quantitative RT-PCR and quantitative real-time PCR

Total RNA was extracted using TRIzol reagent (TaKaRa, China). The first strand cDNAs were synthesized using oligo (dT) primers and MML reverse transcriptase (Promega. China). Specific semi-quantitative RT-PCR primers used to amplify *OsNPF2.2* and *eEF-1a* were as follows: *OsNPF2.2* forward, 5′-GCCGTCGCAGGAGCAAACT-3′; *OsNPF2.2* reverse, 5′-CGAGGAGGAGGCACAAGGTG-3′; *eEF-1a* forward, 5′-AGCCGCTGAGATGAACAA-3′; and *eEF-1a* reverse, 5′-GAGATGGGAACGAAGGGA-3 ′. Quantitative real-time PCR (qPCR) was performed using SYBR Green Premix (TaKaRa) and monitored with the 7500 RT-qPCR system (Applied Biosystems,USA). The primers used for qPCR were as follows: *OsNPF2.2* forward, 5′-GCAGGAGCAAACTAAGCT-3′; *OsNPF2.2* reverse, 5′-TGGGGATGTGGAAGACGGT-3′, *eEF-1a* forward, 5′ -GCACGCTCTTCTTGCTTTC-3′; and *eEF-1a* reverse, 5′-AGGGAATCTTGTCAGGGTTG -3′.

### Identification of the T-DNA-generated mutants *osnpf2.2-1* and *osnpf2.2-2*

Flanking sequence tags (FSTs) for *osnpf2.2-1* and *osnpf2.2-2* were amplified by PCR, and the resulting products were sequenced to confirm the presence of the T-DNA in *OsNPF2.2*. The following primers were used to screen the mutant lines: a, 5′-TGGACTGTGCCACGTGTAAT-3′; b, 5′-CCAGTTGATGTTGCTCTGGA-3′; c, 5′-TTGGGGTTTCTACAGGACGTAAC-3′; d, 5′-ACGGTGTTCCAGGTGATG-3′; and e, 5′-CAGGGTGAAGGTTTAGCAAT-3′. Homozygous lines were screened by PCR using a combination of primers a, b, and c for *osnpf2.2-1* or a combination of primers d, c, and e for *osnpf2.2-2*. Gene expression was confirmed by RT-PCR as described above. Southern blotting was used to examine the T-DNA copy number in the two mutants. Their genomic DNAs were digested with *Xba*I, *Bam*HI and *Hin*dIII and hybridized with a digoxigenin-DNA-labeled hygromycin-resistance gene probe.

### Phenotypic analysis in a paddy field

Rice seeds were surfaced sterilized and germinated in 1/2 Murashige and Skoog media for 10 days; then the seedlings were transferred into an SCBG paddy field for 30 days. Finally, the seedlings were transplanted in the paddy field under normal rice growth conditions and fertilized as for normal rice growth.

### GUS staining for plants grown under normal N conditions or with nitrate induction

Homozygous T_3_ transgenic plants with *pOsNPF2.2-uidA* or with *p35S-uidA* were used for GUS staining. The protocols for GUS staining and section analysis have been described previously^51^. To determine *OsNPF2.2* expression under normal N conditions, plants were grown in a paddy field under normal fertilizer application. For nitrate induction, the rice seedlings were grown in IRRI solution with 1.4 mM KNO_3_ for 10 d and then transferred to IRRI solution with 1.4 mM NH_4_SO_4_ as the N source for NO_3_^-^ deprivation for 1 d. Finally, the plants were transferred back into IRRI solution with 10 mM KNO_3_ to induce GUS activity within 2 h.

### Semi section and transmission electron microscopy analysis

Sample preparation was carried out according to the procedures described by Park *et al*.^57^, Ultra-sections were examined under transmission electron microscopy (JEM-1010 electron microscope; JEOL, Tokyo, Japan). For semi section observation, the 2 µm thick sections were stained with 0.5% toluidine blue and then observed and photographed using a fluorescence microscope (AXIOPLAN 2; Zeiss, Jena, Gemany).

### Transient expression of *OsNPF2.2* in rice protoplasts

To determine the subcellular localization of OsNPF2.2, *p35S-OsNPF2.2-GFP* and *p35S-GFP* were introduced into rice protoplasts. The protocols for rice protoplast preparation and transformation have previously been described^58^. Protoplasts were observed using a confocal laser-scanning microscope (Leica TCS 5 SP5 AOBS), a Leica Microsystem LAS AF, or a Leica DMI6000B inverted fluorescence microscope, as previously described^40^.

### Nitrate content analysis by high-performance liquid chromatography

Rice xylem sap collection was performed according to the protocol described by Tang *et al*^59^, as follows. The rice plants were grown in IRRI solution containing 1.4 mM NH_4_NO_3_ for 8 weeks; then, seedlings were transferred to IRRI solution containing 1.4 mM NH_4_SO_4_ as the N source for NO_3_^−^ deprivation for 1 week. Next, the plants were cut 4 cm above the ground level, the roots were immediately transferred to 10 mM NO_3_^−^, and a preweighed absorbent cotton ball used to collect the xylem exudate was attached to the cut surface and covered with plastic film for 2 h. Next, the collected xylem sap was squeezed from the cotton by using a syringe, and the volume of the exudate was calculated based on the increase in the weight of the cotton. For extraction of total nitrate, the roots and shoots were collected separately, and the nitrate was extracted in boiling distilled H_2_O for 30 min. Nitrate concentration was determined by high-performance liquid chromatography^60^ using a PARTISIT 10 SAX (strong anion exchanger) column (Waterman), with 50 mM KH_2_PO_4_ buffer, pH 3.0, as the mobile phase.

## Supplementary Material

Supplementary Information Supplementary Information
