# Supplementary material for: Disruption of the rice nitrate transporter OsNPF2.2 hinders root-to-shoot nitrate transport and vascular development
Source: Sci Rep. 2015 Apr 29;5:9635. doi: 10.1038/srep09635 (PMC5386202; doi:10.1038/srep09635)
Supplement: Supplementary Information [file srep09635-s1.pdf]

Disruption of the Rice Nitrate Transporter OsNPF2.2 Hinders Root-to-Shoot Nitrate Transport and Vascular Development

Yuge Li[1†], Jie Ouyang[2†], Ya-Yun Wang[3], Rui Hu[1, 4], Kuaifei Xia[1], Jun Duan[1], Yaqin Wang[5], Yi-Fang Tsay[6] & Mingyong Zhang[1*]

[1]*Key Laboratory of South China Agricultural Plant Molecular Analysis and Genetic Improvement & Guangdong Provincial Key Laboratory of Applied Botany, South China Botanical Garden, Chinese Academy of Sciences, Guangzhou 510650, China;* [2]*Rice Institute, Chongqing Academy of Agricultural Sciences, Chongqing 401329, China;* [3]*Department of life science, National Taiwan University, Taipei, Taiwan;* [4]*University of Chinese Academy of Sciences, Beijing 100049, China;* [5]*Guangdong Key Lab of Biotechnology for Plant Development, College of Life Science, South China Normal University, Guangzhou 510631, China; and* [6]*Institute of Molecular Biology, Academia Sinica, Taipei, Taiwan*

[†] These authors contributed equally to this work.

[*] To whom correspondence should be addressed. E-mail zhangmy@scbg.ac.cn, tel. +86 20 3725 2891

Supplementary figure legends

Figure S1. Alignment of the amino-acid sequence for OsNPF2.2 to those of several nitrate and glucosinolate transporters from *Arabidopsis*.

Figure S2. Spatiotemporal expression profile of *OsNPF2.2* in various organs throughout the entire life cycle of rice under natural paddy conditions.

Figure S3. *OsNPF2.2*-RNAi phenotypic copy of the *osnpf2.2* mutants.

(a) Relative expression level of the independent *OsNPF2.2*-RNAi transgenic lines by qRT-PCR analysis. (b-c) Comparison of the seedling growth between the *OsNPF2.2*-RNAi transgenic lines and the wild types (WT), bar = 2cm. (d) Panicle length comparison of the *OsNPF2.2*-RNAi transgenic lines and the wild types (WT), bar = 2cm. (e, f) Statistic analysis of panicle length and seed setting rate of the transgenic lines and the wild types.

Figure S4. Growth retardation of the *osnpf2.2* mutants when low or high levels of nitrate were supplied.

(a-c)The seedlings were grown in IRRI solution for two weeks with 0.2 mM (a), 1.4 mM (b) or 10 mM (c) nitrate as sole N source, bar = 2cm. (d) Statistic analysis of plant height under different concentration of nitrate. One or double asterisks indicate a significant difference ($P < 0.05$ or $P < 0.01$) of plant height (d) compared with the values of the wild types.

Figure S5. Cross sections of various organs from wild-type plants and the *osnpf2.2*

mutants.

Less and disordered arrangement of vascular bundles were observed in culms (a-d, bar: 50 μm), leave blades (e-h, bar = 20 μm), leave sheath (i-l, bar = 50 μm), primary branches (m-p, bar = 10 μm), anthers (q-t, bar = 50 μm), and filaments (u-x, bar = 20 μm) of the *osnpf2.2* mutants, compared with the wild types. WT, wild-type.

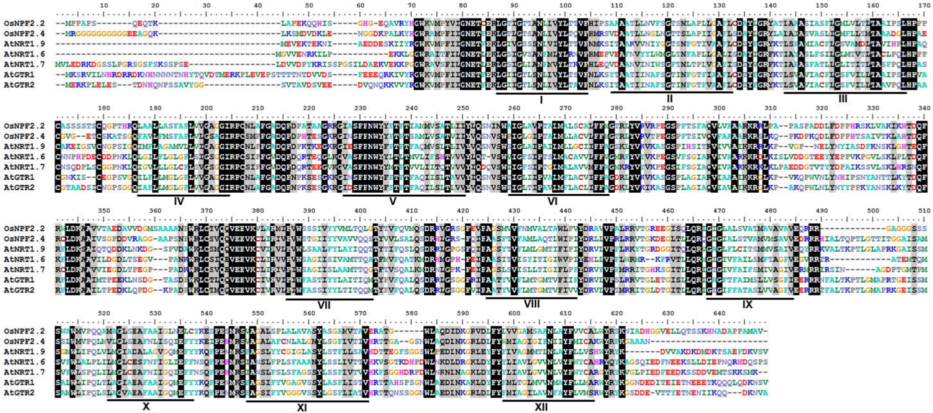
OsNPF2.2
OsNPF2.4
AtNRT1.9
AtNRT1.6
AtNRT1.7
AtGTR1
AtGTR2
I
II
III
IV
V
VI
VII
VIII
IX
X
XI
XII

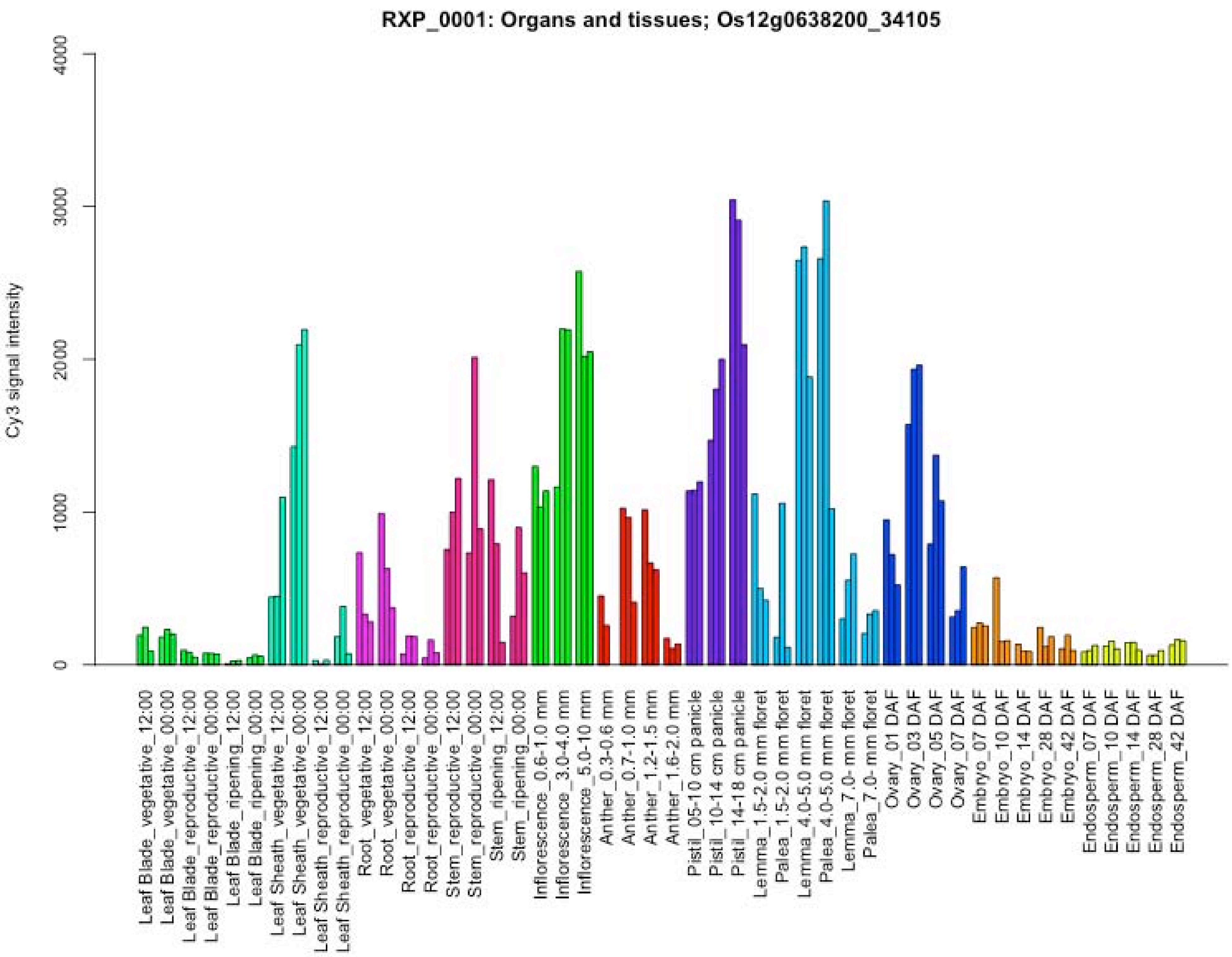
RXP_0001: Organs and tissues; Os12g0638200_34105
Cy3 signal intensity
0
1000
2000
3000
4000
Leaf Blade_vegetative_12:00
Leaf Blade_vegetative_00:00
Leaf Blade_reproductive_12:00
Leaf Blade_reproductive_00:00
Leaf Blade_ripening_12:00
Leaf Blade_ripening_00:00
Leaf Sheath_vegetative_12:00
Leaf Sheath_vegetative_00:00
Leaf Sheath_reproductive_12:00
Leaf Sheath_reproductive_00:00
Root_vegetative_12:00
Root_vegetative_00:00
Root_reproductive_12:00
Root_reproductive_00:00
Stem_reproductive_12:00
Stem_reproductive_00:00
Stem_ripening_12:00
Stem_ripening_00:00
Inflorescence_0.6-1.0 mm
Inflorescence_3.0-4.0 mm
Inflorescence_5.0-10 mm
Anther_0.3-0.6 mm
Anther_0.7-1.0 mm
Anther_1.2-1.5 mm
Anther_1.6-2.0 mm
Pistil_05-10 cm panicle
Pistil_10-14 cm panicle
Pistil_14-18 cm panicle
Lemma_1.5-2.0 mm floret
Palea_1.5-2.0 mm floret
Lemma_4.0-5.0 mm floret
Palea_4.0-5.0 mm floret
Lemma_7.0- mm floret
Palea_7.0- mm floret
Ovary_01 DAF
Ovary_03 DAF
Ovary_05 DAF
Ovary_07 DAF
Embryo_07 DAF
Embryo_10 DAF
Embryo_14 DAF
Embryo_28 DAF
Embryo_42 DAF
Endosperm_07 DAF
Endosperm_10 DAF
Endosperm_14 DAF
Endosperm_28 DAF
Endosperm_42 DAF

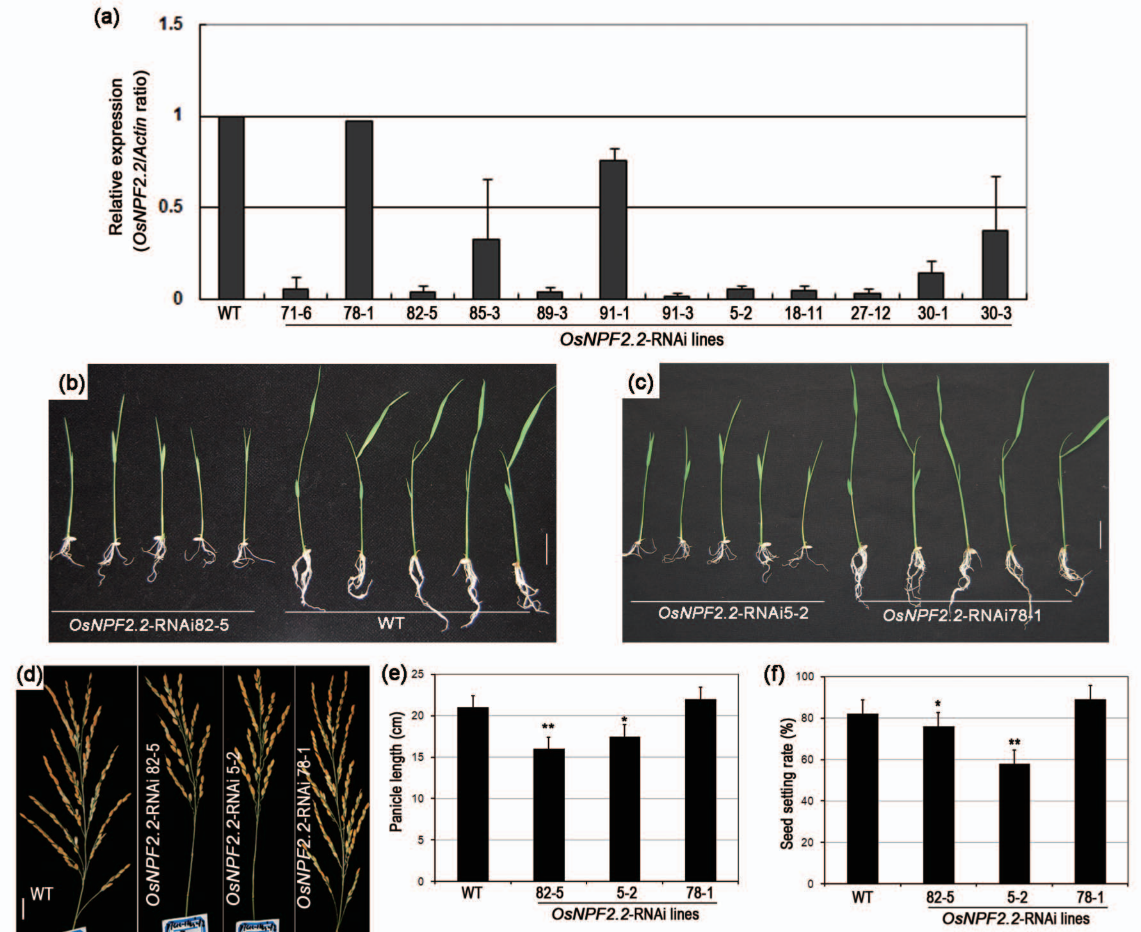

(a)
Relative expression (OsNPF2.2/Actin ratio)
1.5
1
0.5
0
WT
71-6
78-1
82-5
85-3
89-3
91-1
91-3
5-2
18-11
27-12
30-1
30-3
OsNPF2.2-RNAi lines
(b)
OsNPF2.2-RNAi82-5
WT
(c)
OsNPF2.2-RNAi5-2
OsNPF2.2-RNAi78-1
(d)
WT
OsNPF2.2-RNAi 82-5
OsNPF2.2-RNAi 5-2
OsNPF2.2-RNAi 78-1
(e)
Panicle length (cm)
25
20
15
10
5
0
**
*
WT
82-5
5-2
78-1
OsNPF2.2-RNAi lines
(f)
Seed setting rate (%)
100
80
60
40
20
0
*
**
WT
82-5
5-2
78-1
OsNPF2.2-RNAi lines

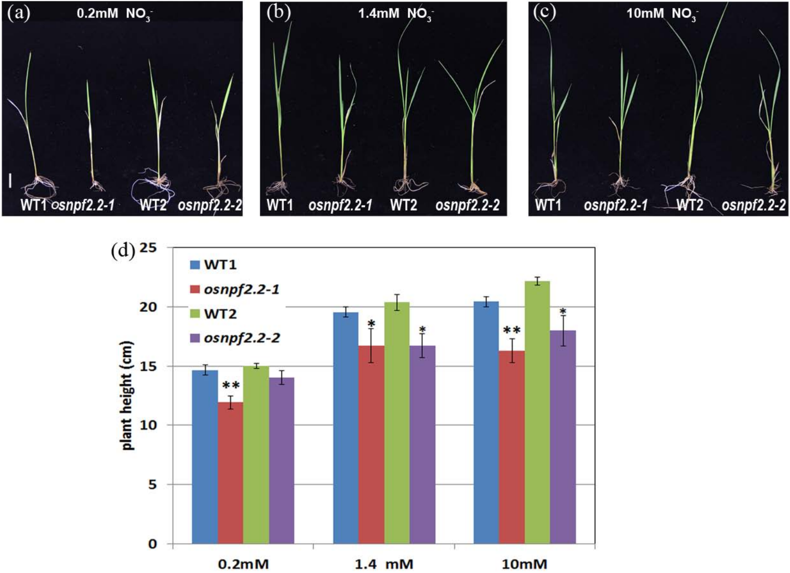
(a)
0.2mM NO_3^-
WT1 osnpf2.2-1 WT2 osnpf2.2-2
(b)
1.4mM NO_3^-
WT1 osnpf2.2-1 WT2 osnpf2.2-2
(c)
10mM NO_3^-
WT1 osnpf2.2-1 WT2 osnpf2.2-2
(d)
WT1
osnpf2.2-1
WT2
osnpf2.2-2
plant height (cm)
0
5
10
15
20
25
**
*
*
**
*
0.2mM
1.4 mM
10mM

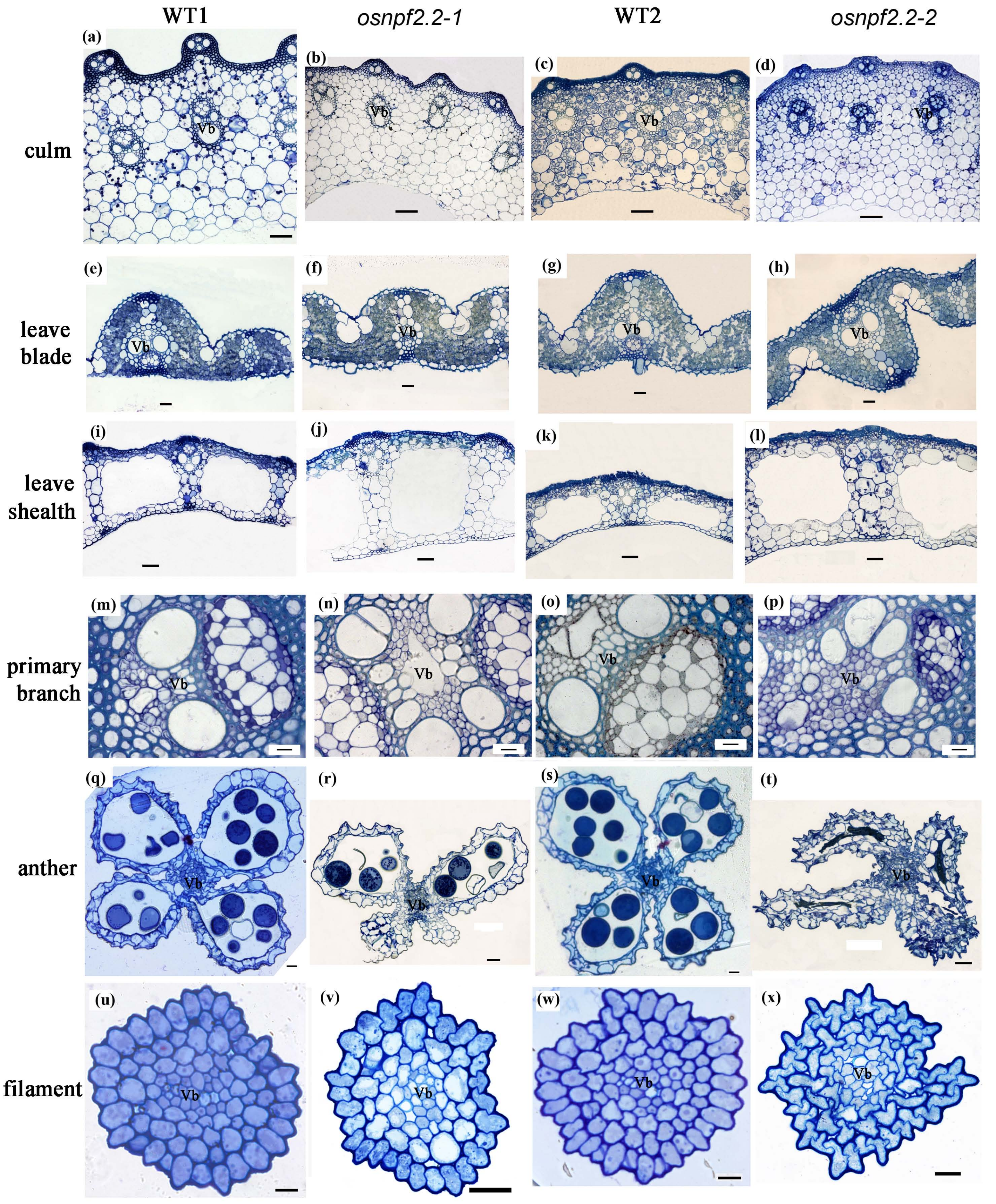

WT1
osnpf2.2-1
WT2
osnpf2.2-2
culm
leave blade
leave shealth
primary branch
anther
filament
(a)
(b)
(c)
(d)
(e)
(f)
(g)
(h)
(i)
(j)
(k)
(l)
(m)
(n)
(o)
(p)
(q)
(r)
(s)
(t)
(u)
(v)
(w)
(x)
Vb
